# *Acinetobacter baumannii*: Mechanisms of antibiotic resistance, quorum sensing regulation, and current therapeutic strategies

**DOI:** 10.1080/21505594.2026.2664980

**Published:** 2026-04-29

**Authors:** Jamil Allen G. Fortaleza, Kevin Smith P. Cabuhat, Christian Joseph N. Ong, Ferdinand A. Mortel, Grace D. Bacalzo, Jose Jurel M. Nuevo

**Affiliations:** a National University Philippines, Manila, Philippines; b Department of Biology, College of Science, De La Salle University, Manila, Philippines; c Basic Education Department, La Consolacion University Philippines, Malolos, Philippines; d College of Medical Technology, Manila Central University, Caloocan, Philippines; e College of Allied Medical Sciences, Wesleyan University-Philippines, Cabanatuan City, Philippines; f College of Medical Laboratory Science, Our Lady of Fatima University, Valenzuela, Philippines

**Keywords:** Biofilm-mediated persistence, carbapenem-resistant *A. baumannii*, horizontal gene transfer, non-antibiotic, multi-drug resistance

## Abstract

*Acinetobacter baumannii* has emerged as a major global pathogen due to extensive resistance to last-resort antimicrobials, a high burden of nosocomial infections, and increasing community-acquired cases. Its adaptability is driven by diverse resistance mechanisms, including β-lactamase production, aminoglycoside-modifying enzymes, efflux pump overexpression, target-site mutations, and lipid A remodeling, all of which limit treatment options and worsen clinical outcomes. Pathogenicity is further enhanced by quorum-sensing systems, particularly AbaI/AbaR, which regulate biofilm formation, virulence, and antimicrobial tolerance. Despite extensive research, resistance, quorum sensing, and therapeutic strategies are often examined separately, limiting mechanistic understanding. This review integrates current evidence on the interplay between resistance evolution, quorum sensing, and biofilm persistence, linking these to therapeutic vulnerabilities. It further evaluates emerging interventions, including optimized antibiotic combinations, immunomodulation, drug repurposing, bacteriophage therapy, and alternative approaches, such as antimicrobial peptides, phytochemicals, nanotechnology, and photodynamic therapy to inform improved treatment strategies.

## Introduction

*Acinetobacter baumannii*, once regarded as a low-virulence organism, has emerged as a major global pathogen due to the rapid dissemination of multidrug-resistant (MDR), extensively drug-resistant (XDR), and pan-drug-resistant (PDR) lineages that severely limit therapeutic options and complicate clinical management [1–4]. The pathogen is a leading cause of healthcare-associated infections, including ventilator-associated and hospital-acquired pneumonia, bloodstream infections, urinary tract infections, meningitis, wound infections, and device-associated infections, predominantly affecting critically ill patients exposed to prolonged hospitalization, invasive procedures, and extensive antimicrobial therapy [5–7]. Its persistence under intense antimicrobial pressure is driven by a complex resistome that encompasses β-lactamase production, including carbapenem-hydrolyzing enzymes, overexpression of resistance-nodulation-division (RND) efflux pumps, target-site modifications, permeability defects, and biofilm-associated tolerance, collectively constraining treatment efficacy [8–12]. Reflecting its clinical impact, carbapenem-resistant *A. baumannii* (CRAb) has been designated by the World Health Organization as a critical priority pathogen, with mortality rates exceeding 60% in intensive care units and reaching up to 84% in ventilator-associated pneumonia caused by XDR strains [4,5,7,13]. As a member of the ESKAPE group, *A. baumannii* exemplifies the accelerating failure of conventional antibiotics and highlights the urgent need for alternative therapeutic and preventive strategies [14–16].

In addition to antimicrobial resistance, the pathogenic success of *A. baumannii* is closely linked to quorum sensing (QS), a population density-dependent regulatory system that coordinates gene expression through diffusible signaling molecules, primarily N-acyl-homoserine lactones (AHLs) [17,18]. QS in *A. baumannii* is mainly mediated by the LuxI/LuxR-type AbaI/AbaR system, which regulates genes involved in biofilm formation, surface-associated motility, virulence factor production, and antimicrobial tolerance, thereby promoting persistence in both environmental and host-associated niches [12,19–24]. QS activity is further shaped by environmental cues, including iron availability, showing how metabolic stress influences virulence regulation [25].

QS-regulated virulence is functionally intertwined with the pronounced biofilm-forming capacity of *A. baumannii*, a defining trait that substantially enhances persistence and tolerance to antimicrobial treatment. A large proportion of clinical isolates exhibits strong biofilm-forming ability, a phenotype more prevalent than in other *Acinetobacter* species, which confers protection through impaired antimicrobial penetration, altered metabolic states, and reduced drug susceptibility [26,27]. Although traditionally associated with hospital environments, *A. baumannii* is increasingly implicated in community-acquired infections, with environmental reservoirs, such as soil, water, plants, animals, food products, and high-contact urban surfaces facilitating transmission between community and healthcare settings [28–30]. Of particular concern, food-derived isolates frequently display robust biofilm formation and multidrug resistance, often carrying plasmid-encoded carbapenemase genes that promote horizontal dissemination of resistance determinants [30,31]. Within clinical settings, biofilm formation enables prolonged survival on abiotic surfaces and medical devices, contributing to central venous catheter-associated bloodstream infections and ventilator-associated pneumonia [6,32,33]. Biofilm-associated cells may also enter dormant or persister states, enhancing survival, clonal expansion, and outbreak potential in hospital environments [34–36]. Key determinants of *A. baumannii* biofilm development includes outer membrane protein A (OmpA), biofilm-associated protein (Bap), chaperone-usher pili (Csu), extracellular polysaccharides, and the BfmS/BfmR two-component regulatory system, which together coordinate adhesion, biofilm maturation, and structural stability [6,26,32,37]. Clinically, biofilm-associated infections are linked to substantially increased mortality, ranging from approximately 35% in bloodstream infections and ventilator-associated pneumonia to nearly 60% in severe community-acquired pneumonia [33,38–40].

In this review, we aim to (i) critically synthesize current knowledge on the molecular mechanisms driving antimicrobial resistance in *A. baumannii*; (ii) examine the role of quorum sensing in regulating virulence, biofilm formation, and antimicrobial tolerance; and (iii) evaluate emerging therapeutic strategies, including antibiotic combinations and non-antibiotic approaches, that target resistance- and QS-associated vulnerabilities to address current treatment limitations and mitigate the global threat posed by these pathogens.

### Collapse of the antibiotic era in *A. baumannii*

*A. baumannii* has developed a highly sophisticated and multilayered resistance architecture that integrates enzymatic drug inactivation, target-site modification, altered membrane permeability, and active efflux, collectively undermining the efficacy of nearly all major antibiotic classes. This coordinated resistome enables rapid adaptation under antimicrobial pressure and has been central to the progressive erosion of effective treatment options. Resistance to β-lactam antibiotics is predominantly mediated by a diverse repertoire of β-lactamases, particularly OXA-type carbapenemases, whereas resistance to aminoglycosides largely arises from the activity of aminoglycoside-modifying enzymes (AMEs). Fluoroquinolone resistance is driven by mutations in DNA gyrase (*gyrA*) and topoisomerase IV (*parC*), frequently acting in concert with the overexpression of resistance-nodulation-division (RND) efflux pumps. Tigecycline resistance similarly reflects a multifactorial process involving enhanced efflux activity, porin loss, and enzymatic inactivation, while resistance to colistin, the last line of defense against multidrug-resistant strains, is primarily associated with lipid A modification or complete loss of lipopolysaccharide (LPS).

In *A. baumannii*, chromosomal mutations remain the dominant mechanism driving colistin resistance. Mutations in the *pmrCAB* operon, particularly within the two-component regulatory genes *pmrA* and *pmrB*, lead to lipid A modifications, such as the addition of phosphoethanolamine (PEtN), which reduces colistin binding and antimicrobial efficacy [41–45] (Silva Nodari et al., 2021; Acharya et al., 2025; Novović & Jovčić, 2023; Vijayakumar et al., 2024; Khoshbayan et al., 2021). In parallel, the inactivation of lipid A biosynthesis genes (*lpxA, lpxC*, and *lpxD*) can result in complete loss of lipopolysaccharide (LPS), conferring high-level resistance [42,44,45]. Resistance mechanisms are heterogeneous across isolates, for instance, insertion sequence – mediated overexpression of *eptA*, particularly via elements, such as ISAba1, can further enhance lipid A modification and resistance levels [44]. Although these chromosomal alterations may impose fitness costs, they can also promote adaptive phenotypes such as enhanced biofilm formation, thereby complicating treatment outcomes and persistence in clinical settings [42].

**Figure 1.**
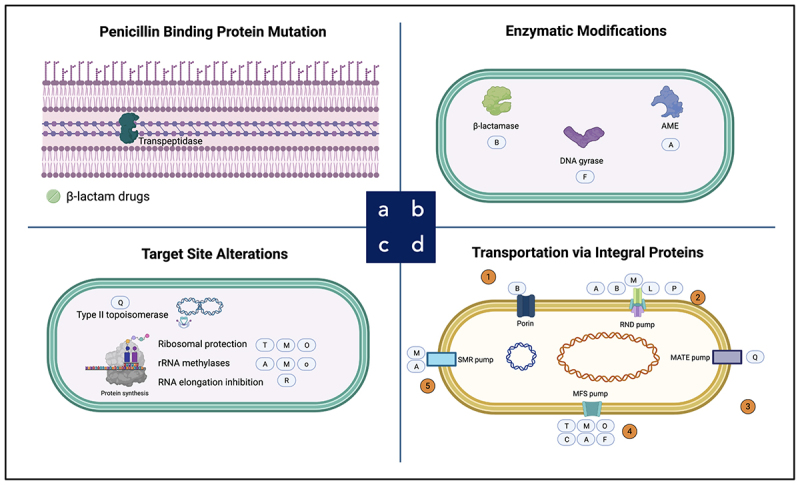
Mechanisms of antibiotic resistance in *A. baumannii*.

Rather than functioning as isolated mechanisms, these resistance determinants operate in an integrated and often synergistic manner, amplifying antimicrobial tolerance and accelerating therapeutic failure. To contextualize this complexity, Table 1 summarizes the principal resistance phenotypes and their associated genetic determinants, while Figure 1 illustrates the coordinated interplay between enzymatic degradation, target alteration, membrane remodeling, and efflux-mediated drug extrusion. Together, these features show how the collapse of the antibiotic era in *A. baumannii* is driven not by single resistance traits, but by a tightly interconnected molecular network that underpins its extraordinary adaptability and clinical threat.

**Table 1.** Major antimicrobial resistance phenotypes and their associated genetic determinants in *Acinetobacter baumannii*.

Resistance mechanism	Class/Subgroup	Representative genes	References
β-Lactam resistance (β-lactamase production)	Class A β-lactamases	*bla*_SCO-1_, *bla*_PER-1_, *bla*_TEM_ variants, *bla*_SHV-5_, *bla*_CTX-M-2/15_ (representatives; see Supplementary File for complete list)	[46–50]
	Class B MBLs	*bla*_NDM-1_, *bla*_IMP-1_, *bla*_VIM_ (representatives; see Supplementary File)	[47,51]
	Class C ADC-type cephalosporinases	*bla*_ADC-1_-*bla*_ADC-268_ (representatives; see Supplementary File)	[47,52,53]
	Class D OXA-type carbapenemases	*bla*_OXA-23_-*bla*_OXA-940_ (representatives; see Supplementary File)	[54–58]
β-Lactam resistance (loss of membrane proteins)	Outer membrane porins	*carO*, *oprD*	[59]
Aminoglycoside resistance (AMEs)	AACs	*aac(3)-Ia*, *aac(6’)-Ib*, *aac(3)-IIa*, *aac(2’)-Ib* (representatives; see Supplementary File)	[60–64]
	ANTs	*ant(2’’)-Ia*, *ant(3’’)-Ia* (representatives; see Supplementary File)	[62,64]
	APHs	*aph(3’)-Ia*, *aph(3’)-VIa*, *aph(3’)-IIb* (representatives; see Supplementary File)	[62–64]
	Adenyltransferases	*aadA1*, *aadB*	[65]
	16S rRNA methylases	*armA*	[61,64]
	SMR efflux pumps	*abeS*	[66]
Fluoroquinolone resistance	DNA gyrase/topoisomerase IV	*gyrA*, *parC*	[67]
	Altered PBPs	*pbp1a-pbp7/8*	[68]
	PMQR	*qnrA*, *qnrB*, *qnrS*, *aac(6’)-Ib-cr*	[69–71]
	RND efflux pumps	*adeABC*, *adeFGH*, *adeIJK*	[72,73]
	MFS efflux pumps	*abaQ*, *abaye2281468*, *cmlA*	[71,74,75]
Tigecycline resistance	RND efflux pumps	*adeB*, *adeFGH*	[76,77]
	MATE efflux pumps	*abeM*	[77]
	Membrane phospholipid metabolism	*plsC*	[78]
	Flavin-dependent monooxygenases	*tet(X)*, *tet(X1-X7)* (representatives; see Supplementary File)	[77,79,80]
Colistin resistance	Lipid A biosynthesis	*lpxA*, *lpxC*, *lpxD*	[81]
	Two-component systems/phosphoethanolamine transferases	*pmrB*, *eptA*	[82]
	Plasmid-mediated colistin resistance	*mcr-4*	[83]

Antibiotic resistance in *A. baumannii* is mediated by four principal mechanisms: (a) Penicillin binding protein mutation, (b) enzymatic modifications, (c) target site alterations, and (d) transportation via integral proteins. Abbreviations: A, aminoglycosides; B, β-lactams; C, chloramphenicol; F, fosfomycin; L, lincosamides; M, macrolides; MATE, multidrug and toxic compound extrusion; MFS, major facilitator superfamily; O, oxazolidinones; P, polymyxins; PBP, penicillin-binding protein; Q, fluoroquinolones; R, rifamycins; RND, resistance-nodulation-division; S, diaminopyrimidines and sulfonamides; SMR, small multidrug resistance family; T, tetracyclines.

#### β-lactam resistance mediated by β-lactamase production

β-Lactam antibiotics have historically been among the most effective antimicrobial agents. However, resistance emerged rapidly following their clinical introduction, underscoring the evolutionary plasticity of bacterial pathogens. In Gram-negative bacteria, including *A. baumannii*, the dominant mechanism of acquired β-lactam resistance is the production of β-lactamases, which are classified into four molecular classes (A-D) based on sequence homology and catalytic mechanism [84]. In *A. baumannii*, the clinical relevance of these enzymes lies not only in their hydrolytic activity but also in their frequent association with mobile genetic elements, which facilitates rapid dissemination and stable expression under antibiotic pressure.

Class A β-lactamases are serine-dependent enzymes that primarily hydrolyze penicillins and extended-spectrum cephalosporins and are widely disseminated via plasmids [84–91]. Clinically important representatives include extended-spectrum β-lactamases (ESBLs) and KPC-type enzymes, which substantially reduce the efficacy of frontline β-lactams and contribute to multidrug-resistant phenotypes in *A. baumannii* and other ESKAPE pathogens [92–94]. Structural flexibility within the active site enables point mutations that expand substrate profiles or reduce susceptibility to inhibitors, complicating therapeutic intervention [95,96]. These adaptive features explain the limited durability of β-lactam-β-lactamase inhibitor combinations and indicate the need for next-generation inhibitors and rational combination therapies.

Class B β-lactamases or metallo-β-lactamases (MBLs), represent one of the most clinically problematic resistance mechanisms in *A. baumannii*. These zinc-dependent enzymes, including the globally prevalent NDM, VIM, and IMP variants, hydrolyze nearly all β-lactams, including carbapenems while sparing monobactams such as aztreonam [90,97–102]. The absence of clinically approved MBL inhibitors, combined with the efficient horizontal transfer of MBL genes, has rendered infections caused by MBL-producing strains extremely difficult to treat, positioning this enzyme class as a major driver of carbapenem failure worldwide [98,103,104].

Class C β-lactamases (AmpC-type), which are typically chromosomally encoded and inducible, play a central role in resistance to cephalosporins and monobactams in *A. baumannii* [46,84,105,106]. Clinically significant ADC variants are often associated with upstream insertion sequences, such as ISAba1, which enhance expression and promote high-level resistance [46,86]. The intrinsic resistance of Class C enzymes to classical β-lactamase inhibitors further limits treatment options and explains the poor performance of many inhibitor-based regimens against AmpC-producing strains [105–109]. These features underscore why AmpC-mediated resistance remains a persistent obstacle in clinical management.

Class D β-lactamases, particularly OXA-type carbapenemases, are the most prevalent carbapenem resistance determinants in *A. baumannii* [85,90,110,111]. Among these, OXA-23, OXA-24/40, and OXA-58 are the most clinically dominant and widely disseminated variants, contributing substantially to carbapenem resistance across hospital and environmental reservoirs [86,112,113]. The structural properties of OXA enzymes, including features such as the β5-β6 loop, facilitate carbapenem hydrolysis while conferring poor susceptibility to the existing inhibitors [114,115]. The dominance of OXA-type carbapenemases explains the limited effectiveness of conventional carbapenems and reinforces the need for alternative agents, optimized combination regimens, and improved diagnostic surveillance to guide therapy [86,94,116–118].

#### Aminoglycoside resistance mediated by aminoglycoside-modifying enzymes

Aminoglycosides remain an important component of combination therapy for severe *A. baumannii* infections due to their rapid bactericidal activity and synergy with β-lactams [119]. However, their clinical utility has been severely compromised by the accumulation of multiple resistance mechanisms. Aminoglycoside resistance in *A. baumannii* is primarily mediated by AMEs, 16S rRNA methyltransferases, and active efflux systems [60,119–121].

Among AMEs, acetyltransferases, nucleotidyltransferases, and phosphotransferases represent the most prevalent and clinically relevant enzyme families, collectively inactivating aminoglycosides through chemical modification that prevents ribosomal binding [60,119–121]. In parallel, 16S rRNA methyltransferases, particularly ArmA, confer exceptionally high-level resistance by directly modifying the ribosomal target site, effectively abolishing aminoglycoside activity across multiple subclasses [61,64,121]. Efflux mechanisms, including the SMR-family transporter AbeS, further reduce intracellular drug accumulation and contribute to multidrug resistance phenotypes [62,119,122].

Importantly, aminoglycoside resistance in *A. baumannii* rarely arises from a single determinant. Instead, the co-occurrence of AMEs, target modification, and efflux systems within individual strains produces cumulative resistance effects that severely limit therapeutic efficacy [120,121,123]. This mechanistic redundancy explains the frequent failure of aminoglycoside monotherapy and highlights the need for combination strategies, efflux inhibition, and the development of structurally modified aminoglycosides capable of evading enzymatic inactivation [61,119,124].

#### Fluoroquinolone resistance via target modification and efflux systems

Fluoroquinolone resistance in *A. baumannii* arises from a coordinated interplay between target-site modification and active efflux, leading to a rapid and often irreversible loss of clinical efficacy. Fluoroquinolones exert their antibacterial activity by inhibiting DNA gyrase and topoisomerase IV, enzymes essential for DNA replication, supercoiling, and chromosome segregation, and have therefore been widely used against *A. baumannii* infections [69]. Resistance most commonly arises from chromosomal mutations in the quinolone resistance0determining regions (QRDRs) of gyrA and parC, which reduce fluoroquinolone binding. Key substitutions, such as Ser83Leu in gyrA and Ser80Leu in parC, are associated with elevated minimum inhibitory concentrations (MICs) of ciprofloxacin and levofloxacin and high-level resistance to nalidixic acid (≥256 µg/mL) [69,125,126]. In contrast, plasmid-mediated quinolone resistance (PMQR) determinants (e.g. qnr genes, aac(6’)-Ib-cr, qepA) confer modest increases in MICs that often remain near susceptibility breakpoints but facilitate the selection of high-level resistance when combined with chromosomal mutations [127–129].

Efflux-mediated resistance acts synergistically with target modification to further diminish intracellular fluoroquinolone concentrations. RND family of efflux pumps plays a central role in this process, with the AdeABC system being the most extensively characterized and clinically relevant [130,131]. Overexpression of AdeABC, often driven by mutations in the regulatory genes *adeR* and *adeS*, markedly enhances fluoroquinolone extrusion and contributes to elevated resistance levels [130,131]. Additional RND transporters, including AdeIJK and AdeFGH, further reduce drug accumulation, while non-RND systems, such as the MFS pump AbeM and SMR transporters broaden the resistance phenotype to multiple antibiotic classes [92,130].

Importantly, fluoroquinolone resistance in *A. baumannii* rarely results from a single genetic event. Instead, the combination of QRDR mutations and efflux pump overexpression produces a cumulative resistance phenotype that severely limits therapeutic responsiveness and reduces the effectiveness of dose escalation or monotherapy. This convergence explains the rapid clinical obsolescence of fluoroquinolones against MDR and XDR *A. baumannii* and highlights the limitations of strategies targeting either target modification or efflux alone. Effective mitigation of fluoroquinolone resistance will likely require integrated approaches that simultaneously address target-site protection, efflux inhibition, and the broader regulatory networks governing multidrug resistance.

#### Tigecycline resistance mediated by efflux, permeability defects, and adaptive responses

Tigecycline is widely used as a last-resort agent for the treatment of MDR *A. baumannii* infections; however, its clinical effectiveness has been increasingly compromised by the emergence of resistance through multifactorial and adaptive mechanisms [132,133]. Central to tigecycline resistance is the overexpression of RND efflux pumps, particularly the AdeABC system, which actively expels tigecycline from the bacterial cell [134–138]. Regulatory mutations in the two-component system *adeRS* and the transcriptional regulator *adeN* lead to constitutive efflux pump expression, thereby sustaining subtherapeutic intracellular drug concentrations. The functional dominance of efflux-mediated resistance is supported by experimental evidence showing that chemical inhibition of efflux activity markedly reduces tigecycline minimum inhibitory concentrations (MICs), directly implicating efflux as the principal determinant of resistance rather than as a secondary contributor [139,140].

Reduced outer membrane permeability further amplifies efflux-driven resistance by limiting tigecycline entry into the bacterial cell [141,142]. Alterations in outer membrane porins decrease antibiotic influx, creating a synergistic resistance phenotype in which active efflux and restricted entry operate in concert to maintain intracellular drug levels below bacteriostatic thresholds, even under high extracellular concentrations. This dual barrier highlights a key limitation of strategies that focus exclusively on efflux inhibition, as restored intracellular accumulation may remain insufficient in the presence of permeability defects. Although enzymatic inactivation of tigecycline has not been definitively characterized, indirect evidence suggests that antibiotic-modifying enzymes may contribute to resistance in a strain-dependent manner, revealing a gap in the current mechanistic understanding and warranting further investigation [143].

Beyond classical resistance determinants, *A. baumannii* exhibits a range of adaptive responses that further enhance the tigecycline tolerance. Biofilm formation reduces antibiotic penetration and promotes localized microenvironments that favor resistance gene expression, while mutations affecting membrane composition, transcriptional regulation, and stress-response pathways contribute to phenotypic tolerance and persistence [144–146]. These overlapping and mutually reinforcing mechanisms explain why tigecycline resistance rarely arises from a single genetic change and why therapeutic strategies targeting only one pathway are often ineffective. Instead, the multifaceted nature of tigecycline resistance underscores the need for a combination of therapies and adjunctive approaches capable of simultaneously disrupting efflux activity, membrane adaptation, and biofilm-associated tolerance.

#### Colistin resistance via lipid a modification and LPS loss

Colistin remains a critical last-line therapy against MDR *A. baumannii* due to its potent activity against Gram-negative bacteria; however, resistance has increasingly emerged through structural alterations of the bacterial outer membrane that diminish antibiotic binding [147–149]. The most direct mechanism involves mutations in the lipid A biosynthesis genes *lpxA*, *lpxC*, and *lpxD*, which can result in partial or complete loss of LPS. This loss substantially reduces the net negative charge of the outer membrane, thereby abrogating colistin’s electrostatic interaction with lipid A and conferring high-level resistance [147–149]. Importantly, LPS-deficient phenotypes often incur significant fitness costs, including impaired growth, reduced motility, attenuated virulence, and compromised biofilm formation, indicating that colistin resistance via LPS loss represents a trade-off between survival under antibiotic pressure and overall pathogenic fitness [147,148]. These costs may partially explain the instability of LPS-deficient strains in antibiotic-free environments but also highlight the capacity of *A. baumannii* to tolerate substantial physiological disruption when under strong selective pressure.

In addition to complete LPS loss, *A. baumannii* frequently employs lipid A modification as a more adaptive and clinically relevant resistance strategy. Two-component regulatory systems, particularly PmrAB and PhoPQ, modulate the expression of enzymes responsible for the addition of cationic moieties, such as phosphoethanolamine (pEtN) to lipid A [150–152]. These modifications reduce the negative surface charge of the bacterial membrane, weakening colistin binding while largely preserving membrane integrity and cellular viability. Among these pathways, PmrAB-mediated regulation represents the dominant chromosomal mechanism of colistin resistance in *A. baumannii*, with activating mutations leading to constitutive lipid A modification and stable resistance phenotypes [147,148,150]. In contrast, PhoPQ-mediated responses appear more context-dependent, functioning as adaptive mechanisms under specific environmental stresses such as low magnesium conditions [152]. The preferential selection of lipid A modification over complete LPS loss in clinical isolates underscores the evolutionary advantage of resistance mechanisms that balance antimicrobial evasion with retained fitness and virulence.

Of particular concern is the emergence of plasmid-mediated *mcr* genes, especially *mcr-1*, which encode phosphoethanolamine transferases capable of modifying lipid A independently of chromosomal regulatory systems [153–155]. Unlike chromosomal mutations, *mcr*-mediated resistance facilitates horizontal gene transfer across bacterial species and ecological niches, greatly expanding the reservoir of colistin resistance determinants. Although *mcr* genes are less prevalent in *A. baumannii* than in Enterobacterales, their increasing detection in environmental, animal, and clinical isolates raises serious concerns regarding the long-term viability of colistin as a last-resort therapy [153–155]. The global dissemination of *mcr* genes highlights the fragility of reliance on polymyxins and underscores the urgent need for integrated surveillance, antimicrobial stewardship, and the development of alternative therapeutic strategies that do not depend on outer membrane disruption alone [156,157].

In contrast, plasmid-mediated *mcr* genes, such as *mcr-4*, are extremely rare in *A. baumannii* and have been predominantly reported in environmental or food-associated isolates rather than clinical settings [83] (Gelbíčová et al., 2019). When present, *mcr* determinants typically confer only a low-level colistin resistance, rendering their direct contribution to therapeutic failure less significant than chromosomal mutations [42,43]. Consequently, chromosomal mechanisms remain the primary drivers of clinically relevant colistin resistance in *A. baumannii*. Nevertheless, the emergence of plasmid-mediated *mcr* genes is of particular concern due to their capacity for horizontal transfer across bacterial species and ecological niches. These genes encode phosphoethanolamine transferases that modify lipid A independently of chromosomal regulatory systems, thereby enabling resistance dissemination beyond clonal expansion. Although *mcr* genes are far less prevalent in *A. baumannii* than in Enterobacterales, their detection in environmental, animal, and occasional clinical isolates highlights the potential for wider spread, underscoring the need for integrated surveillance, antimicrobial stewardship, and the development of alternative therapeutic strategies that do not rely solely on outer membrane disruption [83,158,159].

#### Role of plasmids in the dissemination of antimicrobial resistance in *A. baumannii*

Plasmids represent a critical and highly efficient mechanism driving the dissemination of antimicrobial resistance in *A. baumannii*, functioning as mobile genetic platforms that facilitate horizontal gene transfer and the accumulation of multiple resistance determinants within single bacterial hosts. In the context of quinolone resistance, plasmid-mediated quinolone resistance (PMQR) genes, including *qnrB*, *qnrS*, and *aac(6’)-Ib-cr*, have been identified in clinical isolates, with *aac(6’)-Ib-cr* generally more prevalent than *qnrB* [160,161]. Other PMQR determinants, such as *qnrA*, *qnrC*, *qnrD*, and *qepA*, are detected less frequently, suggesting a more limited but still relevant epidemiological contribution [160,161]. These genes are commonly embedded within conjugative plasmids and associated mobile genetic elements, including integrons and transposons, which enhance their capacity for inter- and intra-species dissemination [162].

Functionally, PMQR determinants alone confer only low-level resistance to quinolones; however, their clinical significance lies in their ability to act as facilitators of resistance evolution. By lowering susceptibility thresholds, these plasmid-borne genes promote the selection and stabilization of high-level resistance through subsequent chromosomal mutations in the quinolone resistance – determining regions (QRDRs). This synergistic interaction is further amplified by intrinsic resistance mechanisms, particularly efflux systems, such as AdeABC, which often exert a dominant influence on clinically relevant resistance phenotypes [160,162,163]. Thus, plasmids do not merely transfer resistance genes but actively shape the evolutionary trajectory of antimicrobial resistance in *A. baumannii*.

A similar plasmid-driven paradigm is evident in colistin resistance, particularly through the emergence of *mcr* genes. Notably, *mcr-1* is frequently associated with IncX4 plasmids, which are characterized by high transfer efficiency and the capacity to co-harbor additional resistance determinants, such as *qnrVC5*, thereby enabling the simultaneous dissemination of resistance to multiple antibiotic classes [164,165]. The plasmid-mediated nature of *mcr-1* facilitates its horizontal spread across bacterial populations and ecological niches, distinguishing it from chromosomal resistance mechanisms that are typically confined to clonal expansion. When combined with other resistance determinants, including β-lactamases and efflux pump systems, the plasmid-borne genes substantially reinforce the multidrug-resistant phenotype of *A. baumannii* and contribute to the persistence of difficult-to-treat infections [164,165].

### QS in *A. baumannii*

**Figure 2.**
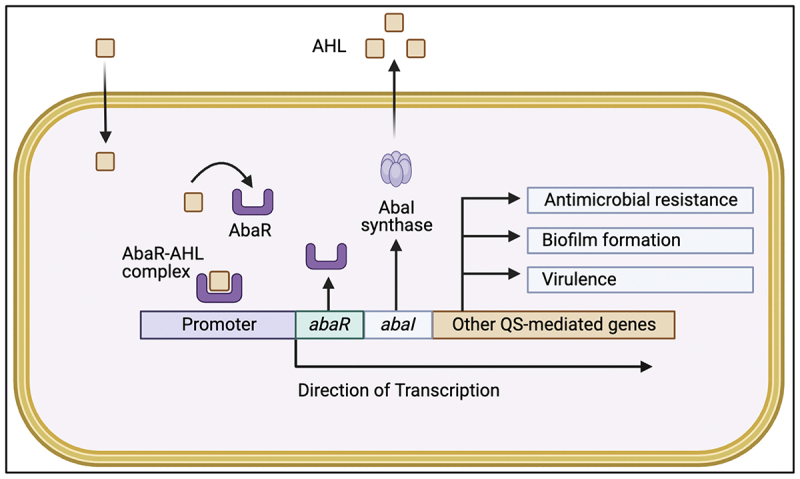
QS-mediated regulation of antimicrobial resistance and biofilm formation in *A. baumannii*.

QS has emerged as a central regulatory system coordinating adaptive behaviors that contribute to persistence and treatment failure in *A. baumannii*. In this pathogen, QS is predominantly mediated by the AbaI/AbaR circuit, a LuxI/LuxR-type system that employs N-acyl homoserine lactone (AHL) signaling to regulate gene expression in a population density – dependent manner [166–168]. AbaI synthesizes diffusible AHL molecules that accumulate as bacterial density increases, while AbaR functions as the cognate transcriptional regulator. Upon reaching a threshold concentration, AHL binding to AbaR triggers transcriptional activation of QS-responsive genes, including positive feedback on *abaI*, thereby amplifying signal output and synchronizing population-wide responses. As depicted in Figure 2, this regulatory loop links environmental sensing to coordinated modulation of antimicrobial tolerance, biofilm formation, and virulence-associated traits rather than acting as a simple on-off switch.

Accumulating experimental evidence supports a direct role for QS in modulating antimicrobial resistance in *A. baumannii*. AHL-deficient mutants lacking *abaI* display increased susceptibility to multiple antibiotic classes, including β-lactams such as meropenem and piperacillin, while exogenous supplementation with N-3-hydroxy-dodecanoyl-homoserine lactone (N-3-OH-C12-HSL) restores resistance phenotypes [166,169]. Similarly, deletion of *abaI* or *abaR* leads to significant reductions in minimum inhibitory concentrations for aminoglycosides and β-lactams, including carbapenems, with resistance reestablished upon genetic complementation, demonstrating a causal link between QS signaling and antimicrobial tolerance [166]. These findings indicate that QS does not merely correlate with resistance but actively modulates resistance expression, likely through indirect regulation of efflux systems, membrane-associated factors, and stress response pathways rather than direct control of resistance genes.

QS regulation is also tightly coupled to biofilm formation, a defining feature of *A. baumannii* persistence. Biofilm development proceeds through coordinated stages of surface attachment, microcolony formation, maturation, and dispersal, supported by an extracellular polymeric substance (EPS) matrix composed of polysaccharides, proteins, extracellular DNA (eDNA), lipids, and enzymes [20,170,171]. During maturation, QS-regulated components, such as poly-β-1,6-N-acetylglucosamine (PNAG), biofilm-associated protein (Bap), and outer membrane protein A (OmpA) enhance structural stability, nutrient retention, and tolerance to antimicrobial stress [172–174]. Importantly, QS-driven biofilms create microenvironments that facilitate horizontal gene transfer at rates reported to be up to 700-fold higher than in planktonic populations, accelerating the dissemination of antimicrobial resistance determinants and reinforcing the resilience of biofilm-associated communities [166,168].

Targeting QS represents a compelling antivirulence strategy that differs fundamentally from conventional antibiotic approaches. By disrupting AHL synthesis or AbaR-mediated signal transduction, QS inhibition aims to attenuate resistance expression, biofilm formation, and virulence without directly impairing bacterial growth. This strategy may reduce selective pressure for classical resistance mechanisms and preserve antibiotic efficacy when used as an adjunct therapy. Experimental studies demonstrating restored antibiotic susceptibility and impaired biofilm formation following QS disruption support the translational potential of this approach [25,166,175]. Moreover, QS inhibitors could synergize with existing antimicrobials by dismantling population-level tolerance mechanisms that are inaccessible to bactericidal agents alone. As resistance in *A. baumannii* increasingly arises from coordinated regulatory networks rather than single genetic determinants, targeting QS offers a rational pathway to weaken pathogenic fitness and persistence while minimizing the evolutionary incentives for resistance development.

In *Acinetobacter baumannii*, quorum sensing (QS) functions as a master regulator of virulence, orchestrating biofilm formation as a critical determinant of bacterial survival and antibiotic tolerance [167–169]. The AbaI/AbaR system produces and senses N-acyl homoserine lactones (AHLs), which accumulate with increasing bacterial density and trigger the transcription of downstream biofilm-associated genes. Importantly, QS does not directly constitute the biofilm structure but regulates key effectors involved in its formation. Among these, the *pgaABCD* operon directs the synthesis of poly-β-1,6-N-acetylglucosamine (PNAG), the primary extracellular polymeric substance that scaffolds early biofilm architecture, with PgaC and PgaD polymerizing N-acetylglucosamine and PgaA and PgaB mediating transport and modification for matrix incorporation [170–174]. As the biofilm matures, the biofilm-associated protein (Bap), characterized by multiple immunoglobulin-like repeats, contributes to structural stability, cell-to-cell aggregation, and adhesion to host or abiotic surfaces, with its expression likewise modulated by QS signaling [176–180]. Thus, QS, PNAG, and Bap operate within a coordinated regulatory-structural hierarchy, in which QS acts as an upstream signaling system that controls the expression of biofilm effectors, linking population-level communication to biofilm development, persistence, and multidrug resistance [168,181,182].

### Biofilm formation as a critical virulence factor in *A. baumannii*

**Figure 3.**
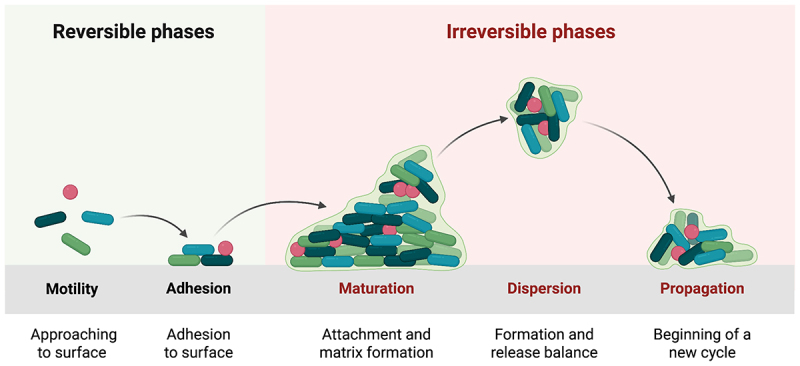
Stages of biofilm formation in *A. baumannii.*

Biofilm formation is a central virulence trait in *A. baumannii*, enabling persistence in hostile clinical environments and contributing substantially to treatment failure. Biofilms are structured bacterial communities embedded within a self-produced extracellular matrix composed primarily of polysaccharides, proteins, and extracellular DNA, which collectively impede antibiotic penetration and protect bacteria from host immune defenses [183–191]. This protective niche promotes chronic infection, often necessitating prolonged or intensified antimicrobial therapy, particularly in ventilator-associated pneumonia and bloodstream infections. In *A. baumannii*, biofilm development proceeds through well-defined stages, initial attachment, microcolony formation, maturation, and dispersal, each coordinated by a network of structural and regulatory determinants that stabilize community architecture and enhance survival (Figure 3).

Biofilm formation in *A. baumannii* is mediated by a limited but functionally diverse set of key factors that collectively govern surface attachment, matrix integrity, and long-term persistence (Table 2). The biofilm-associated protein Bap (*bap-Ab*) promotes intercellular adhesion and biofilm maturation on both biotic and abiotic surfaces [167,168]. Extracellular matrix stability is reinforced by poly-β-(1,6)-N-acetylglucosamine (PNAG), synthesized by the *pgaA – D* locus, which facilitates cell – cell cohesion and maintains biofilm thickness [194,195]. The chaperone – usher pili system CsuA/BABCDE, regulated by the BfmRS and GacSA systems, is essential for initial attachment and biofilm establishment on abiotic surfaces, such as medical devices [172,197]. In parallel, outer membrane protein A (OmpA) supports host cell adhesion, contributes to biofilm development, and enhances cytotoxicity during infection [199]. Notably, certain resistance determinants, including the β-lactamase PER-1, extend their functional role beyond antibiotic hydrolysis by promoting surface adherence and biofilm formation, thereby reinforcing persistence under antimicrobial pressure [196]. Quorum sensing integrates these processes by synchronizing the population-level regulation of biofilm formation, motility, virulence expression, horizontal gene transfer, and antimicrobial resistance [167,200].

**Table 2.** Genes associated with biofilm formation in *A. baumannii*.

Effectors	Gene Determinants	Functions	References
*A. baumannii* biofilm-associated protein (BAP)	*bap*	BAP promotes intercellular adhesion, enabling biofilm maturation, structural stability, and enhanced host cell adherence.	[177,192]
Poly-β-(1,6)-N-acetylglucosamine (PNAG)	*pgaA*, *pgaB*, *pgaC*, *pgaD*	PNAG mediates intercellular adhesion, reinforces the structural integrity of the biofilm matrix, and sustains biofilm thickness, thereby promoting biofilm stability and resilience.	[193,194]
β-lactamase PER1	*bla*_PER-1_	Beyond antibiotic hydrolysis, *bla*_PER-1_ contributes to surface adhesion and biofilm establishment, enhancing bacterial persistence on both host tissues and abiotic surfaces.	[195]
CsuA/BABCDE chaperone-usher pili assembly system	*csuA*, *csuB*, *csuC*, *csuD*, *csuE*,	The Csu pili system facilitates pilus biogenesis and surface attachment, promoting robust biofilm formation and long-term colonization on abiotic substrates through coordinated regulation of assembly and structural maintenance.	[196,197]
BfmRS Two-Component System:	*bfmRS* (*bfmR* and *bfmS*)	*bmfRS* gene system promotes early biofilm formation in *A. baumannii* by upregulating the chaperone-usher secretion system, enabling pili-mediated twitching motility and enhanced surface attachment.	[197]
Outer membrane protein A (OmpA)	*ompA*	OmpA mediates adhesion to host cells, supports biofilm development, and contributes to virulence by promoting invasion and cytotoxic effects on eukaryotic cells.	[198]
QS	*abaR*, *abaM*, *abaI*	QS coordinates population-level behaviors through autoinducer signaling, regulating biofilm formation, expression of virulence determinants, motility, horizontal gene transfer, and antimicrobial resistance.	[177,199]

Within biofilms, *A. baumannii* adopts additional survival strategies that further complicate eradication. Biofilm-associated cells frequently enter dormant or persister states, exhibiting reversible antibiotic tolerance that is particularly pronounced in extensively drug-resistant strains [198,201]. Experimental studies consistently show that biofilm-forming clinical isolates survive antimicrobial exposure at significantly higher rates than planktonic populations, underscoring the limitations of conventional antibiotic regimens. These features highlight the need for alternative therapeutic approaches targeting biofilm-specific vulnerabilities, including disruption of the extracellular matrix, inhibition of biofilm-associated regulatory networks, and elimination of persister cells. Adjunctive strategies such as antimicrobial peptides, bacteriophage-based therapies, and high-throughput screening of anti-biofilm compounds represent promising avenues to mitigate biofilm-mediated resistance and reduce the burden of MDR and XDR *A. baumannii* infections in healthcare settings [190,198,201].

## Current therapeutic strategies

**Figure 4.**
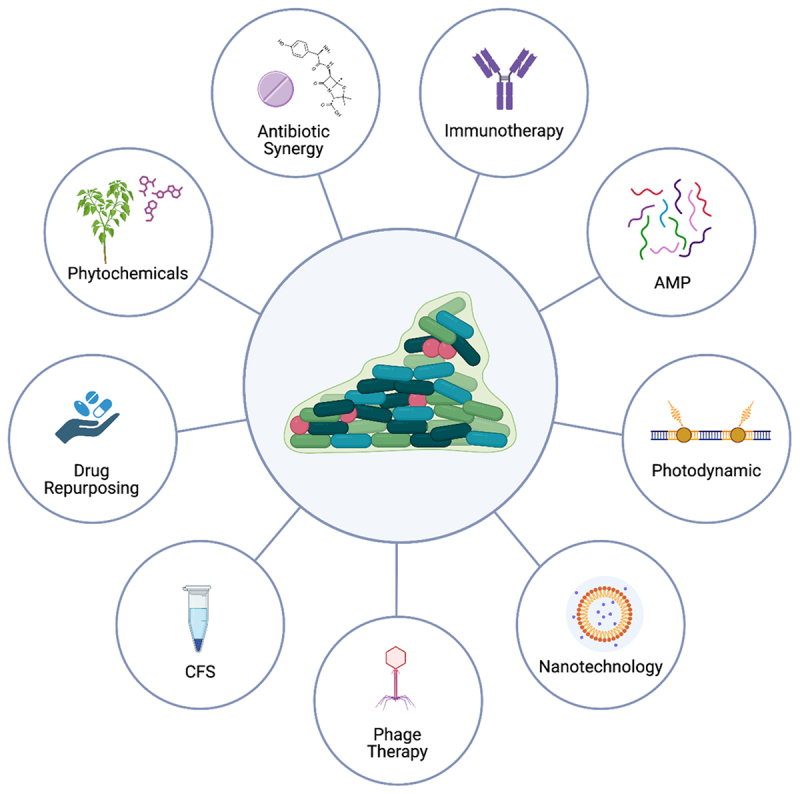
Current therapeutic strategies in combating *A. baumannii*.

*A. baumannii* remains difficult to treat because AMR and persistence are tightly linked: β-lactamase activity, efflux overexpression, permeability defects, and biofilm-associated tolerance act together to limit effective drug exposure at infection sites. As a result, therapeutic success depends less on whether a strategy shows *in vitro* activity and more on whether it can achieve adequate exposure in biofilm-influenced infections, avoid dose-limiting toxicity, and reduce selective pressure for resistance. Figure 4 illustrates the major therapeutic classes currently explored against *A. baumannii*, while Table 3 integrates these approaches according to their primary clinical role, mechanistic value, and translational limitations. Together, they highlight that optimized antibiotic regimens provide the most immediate clinical benefit, whereas delivery-enabled and non-antibiotic strategies, including biofilm-active and preventive approaches, are essential complements for durable control of multidrug-resistant infections.

**Table 3.** Comparative overview of therapeutic and adjunct strategies against multidrug-resistant and biofilm associated *A. baumannii* infections.

Strategy class	Primary clinical role	Core value (why it matters)	Evidence strength (typical)	Best-fit use case	Key limitations/bottlenecks
**Rational antibiotic combinations**	**Treatment**	Rapid bacterial killing; can restore activity via complementary mechanisms	Clinical + observational + *in vivo*	ICU pneumonia, bacteremia, device infections when rapid control is needed	Toxicity (esp. polymyxins), PK/PD complexity, resistance emerging under therapy
**Drug repurposing**	**Adjunct/antibiofilm**	Targets non-classical pathways (e.g. iron metabolism, adhesion), can reduce virulence/biofilm	Mostly *in vitro* + limited *in vivo*	Add-on to antibiotics to weaken biofilm and reduce bacterial fitness	Variable potency in A. baumannii, dosing feasibility at infection site, unclear clinical endpoints
**Nanotechnology/nano-delivery**	**Adjunct/delivery platform**	**Mechanism diversity**: penetration of EPS + direct killing (ions/ROS) + delivery of drugs	*in vitro* + *in vivo* (variable), early translational	Biofilm-heavy infections; local delivery (wounds, devices)	Safety/biocompatibility, aggregation *in vivo*, regulatory complexity, scale-up
**Photodynamic therapy (PDT)**	**Local treatment/device-surface control**	**Biofilm-active** killing via ROS; less tied to classical resistance	*in vitro* + some *in vivo*/local models	Wounds, surfaces, catheters, topical/localized infections	Limited depth penetration, device/light logistics, standardization of dose/light/photosensitizer
**Phage therapy** (phage alone or phage–antibiotic synergy)	**Targeted treatment/adjunct**	High specificity; strong antibiofilm potential; can exploit fitness trade-offs of resistance	Preclinical strong; early clinical/compassionate use	Personalized or cocktail use for MDR/XDR, especially biofilm-associated infections	Host-range matching, resistance to phage, manufacturing/QC, regulatory pathways, timing/delivery
**Cell-free supernatants/probiotics-derived products**	Adjunct (anti-biofilm + immunomodulatory)	Biofilm gene modulation + potential inflammation dampening	Early-stage (mostly *in vitro*)	Supportive adjunct concept, especially where inflammation contributes to damage	Standardization (composition varies), mechanism attribution, limited *in vivo* validation
**Phytochemicals**	Adjunct/anti-virulence/antibiofilm	Mechanism diversity (membranes + QS/biofilm genes + metabolism); synergy with antibiotics	*in vitro* strong; *in vivo* variable	Combination therapy to reduce biofilm/virulence and lower MICs	Bioavailability, reproducibility of extracts, PK/PD gaps, toxicity at effective doses
**Antimicrobial peptides (AMPs)**	**Adjunct or alternative killing strategy**	**Mechanism diversity**: membrane disruption + regulatory/metabolic rewiring; often biofilm-active	Preclinical; formulation-dependent	Biofilm infections, topical/local delivery; combination regimens	Stability, toxicity, cost, delivery (proteases, serum binding)
**Immunotherapy**	**Prevention + antibiotic-sparing**	Reduces infection incidence/severity; supports clearance; may synergize with antibiotics	Preclinical promising; limited clinical validation	High-risk populations, outbreak settings, adjunct passive immunization	Antigenic heterogeneity, strain coverage, manufacturing cost, need for robust clinical trials/endpoints

## Antibiotic combination therapy

Antibiotic combination therapy remains the most immediately actionable strategy for managing multidrug-resistant *A. baumannii*, particularly in severe infections where biofilm-associated tolerance renders monotherapy unreliable [198]. The clinical rationale for the combinations is fundamentally mechanistic rather than empirical: membrane-active agents increase intracellular antibiotic exposure, while β-lactam/β-lactamase inhibitor pairs restore target engagement in β-lactamase-rich backgrounds. Polymyxins (colistin or polymyxin B) combined with carbapenems exemplify this principle, as outer-membrane disruption by polymyxins enhances carbapenem penetration and activity [202]. This strategy is particularly relevant in ventilator-associated pneumonia, bloodstream infections, and device-associated disease, where biofilms limit effective antibiotic concentrations at the infection site [203]. However, the clinical utility of polymyxin-based regimens is constrained by nephrotoxicity, neurotoxicity, and the rapid selection of polymyxin resistance under suboptimal exposure, underscoring the importance of pharmacokinetic optimization and careful patient selection [204].

Recent therapeutic advances have reframed combination therapy from a salvage approach to a strategy of rational susceptibility reconstruction. Cefiderocol exploits bacterial iron uptake pathways to enhance penetration into Gram-negative cells and biofilm-adjacent niches, partially circumventing conventional resistance mechanisms [204–206]. Similarly, the sulbactam – durlobactam combination restores β-lactam activity by simultaneously inhibiting β-lactamases and targeting penicillin-binding proteins, offering a resistance-informed route to reengage essential cell wall synthesis pathways [207]. Collectively, these developments emphasize that the most defensible near-term strategy against MDR *A. baumannii* is not escalation of antibiotic number but optimization of exposure and target engagement, ideally integrated with adjunctive approaches that disrupt biofilms or support host immunity in device- or surface-associated infections.

### Emerging β-Lactam/β-lactamase inhibitor combinations in *Acinetobacter baumannii*

The development of β-lactam/β-lactamase inhibitor (BL/BLI) combinations represents a critical advancement in the management of multidrug-resistant (MDR) Gram-negative infections, particularly carbapenem-resistant *Acinetobacter baumannii* (CRAB). β-lactam antibiotics target penicillin-binding proteins (PBPs) to disrupt bacterial cell wall synthesis; however, their efficacy is frequently compromised by β-lactamases that hydrolyze these agents [208–210]. BLIs restore antibiotic activity by inhibiting these enzymes, thereby enabling effective target engagement and bactericidal action [211–213]. This synergy forms the mechanistic basis for BL/BLI development as a strategy to counter enzymatic resistance.

Among currently available therapies, sulbactam-based combinations remain the most clinically relevant for *A. baumannii*, largely due to the intrinsic antibacterial activity of sulbactam. The sulbactam – durlobactam combination has demonstrated strong efficacy against CRAB and has advanced to clinical use, although data beyond respiratory infections remain limited [214–216]. Similarly, ampicillin/sulbactam continues to be utilized in combination regimens, showing synergy with agents, such as ceftazidime/avibactam. However, the need for higher doses in strains harboring metallo-β-lactamases, such as NDM-1, highlights ongoing resistance challenges [217]. These findings indicate that while sulbactam-based therapies remain central, their effectiveness is increasingly constrained by evolving resistance mechanisms.

Newer BL/BLI combinations have been designed to broaden the spectrum of β-lactamase inhibition and improve activity against diverse MDR pathogens. Agents such as ceftazidime/avibactam, cefepime/enmetazobactam, and cefepime/taniborbactam exhibit enhanced activity against ESBL-producing organisms; however, their efficacy against *A. baumannii* remains inconsistent due to intrinsic resistance factors such as metallo-β-lactamase production and reduced membrane permeability [218–220]. The observed synergy between ceftazidime/avibactam and sulbactam suggests that rational combination strategies may help overcome these limitations, emphasizing the importance of mechanism-informed therapy [217,221].

Emerging BL/BLI agents further expand this approach by incorporating dual mechanisms of action. Combinations such as cefepime/zidebactam and cefepime/nacubactam simultaneously inhibit β-lactamases and target PBPs, enhancing the antibacterial efficacy [222]. Investigational agents, including sulbactam/ETX2514 and LN-1–255–based combinations, demonstrate broad-spectrum activity in preclinical models [223,224]. Despite these advances, clinical effectiveness remains context-dependent and limited by persistent resistance mechanisms. Overall, BL/BLI combinations represent a promising yet evolving strategy, requiring continued optimization and clinical validation to address the complex resistance landscape of *A. baumannii*, with further details summarized in Table 4.

**Table 4.** Emerging β-lactam/β-lactamase inhibitor combinations against multidrug-resistant *Acinetobacter baumannii.*

Combination	Target Pathogen(s)	Outcomex	Clinical Development Status/Limitations	References
Sulbactam/Durlobactam	CRAB	High *in vitro* and *in vivo* efficacy; synergistic activity with imipenem-cilastatin	Approved; limited data beyond respiratory infections	[214–216]
Cefepime/Enmetazobactam	ESBL-producing Gram-negatives	Broad-spectrum activity; effective against *Pseudomonas* and Enterobacterales	Phase III ongoing; limited activity data for *A. baumannii*	[225]
Cefepime/Taniborbactam	MDR Gram-negatives	Broad inhibition of serine β-lactamases	Preclinical; no specific data for *A. baumannii*	[222]
Cefepime/Zidebactam	MDR Gram-negatives	Dual mechanism: β-lactamase inhibition and PBP2 targeting	Preclinical; limited clinical data	[222]
Cefepime/Nacubactam	MDR Gram-negatives	Potent *in vitro* activity; targets PBP2 to enhance antibacterial effect	Preclinical; limited clinical validation	[222]
Ampicillin/Sulbactam	CRAB	Synergistic with ceftazidime/avibactam; effective against resistant strains	Requires high doses for NDM-1-producing isolates	[217]
Ceftazidime/Avibactam	CRAB (limited)	Synergistic with sulbactam; reduces bacterial burden	Limited efficacy against metallo-β-lactamase producers	[217]
Sulbactam/ETX2514	MDR *A. baumannii*	Broad-spectrum synergy across multiple β-lactamase classes	Preclinical; no clinical trial data available	[223]
Cefotaxime/Sulbactam	ICU-associated pathogens	Effective against nosocomial infections, including *A. baumannii*	Limited global availability; primarily regional data	[226–228]
LN-1–255–based combinations	CRAB	Reduces resistance rates to carbapenems and cefepime	Preclinical; no clinical trial data available	[224]

## Repurposing existing drugs

Drug repurposing is attractive because it exploits established safety profiles and pharmacokinetic data; however, its clinical relevance against *A. baumannii* is contingent on whether repurposed agents target pathogen-specific vulnerabilities at concentrations achievable at infection sites [229]. Statins highlight the limitations of this approach. Although antimicrobial and anti-virulence effects have been reported [230,231], their activity against *A. baumannii* is inconsistent, with atorvastatin showing minimal inhibitory effects against XDR clinical isolates and laboratory strains [232]. These findings underscore that broadly pleiotropic host-directed or membrane-modifying effects do not necessarily translate into meaningful antibiofilm or antibacterial activity against this pathogen. Moreover, repurposed drugs administered at subinhibitory concentrations may inadvertently impose selective pressure, potentially promoting tolerance or resistance rather than therapeutic benefit [233].

In contrast, repurposed compounds that align more closely with *A. baumannii* persistence biology demonstrate greater translational promise. Farnesol exhibits robust antibiofilm and biofilm-detachment activity with a favorable resistance-sparing profile, suggesting efficacy beyond growth inhibition alone [229,234]. Gallium nitrate similarly targets iron acquisition, a metabolic dependency tightly linked to survival and biofilm maintenance and has been shown to inhibit bacterial growth and biofilm formation in serum, while destabilizing mature biofilms at higher concentrations [235]. Taken together, these observations indicate that repurposed agents are most effective when deployed as adjunctive therapies, functioning to weaken biofilm integrity or metabolic resilience rather than replacing antibiotics outright. Their strategic value lies in lowering the biofilm barrier and potentially reducing antibiotic dose intensity or duration, rather than serving as stand-alone antimicrobials.

Nanotechnology is best viewed as a solution to the exposure limitations imposed by biofilm architecture rather than as a stand-alone antimicrobial strategy. Nanoparticles can penetrate the EPS, concentrate antimicrobial payloads at the biofilm–bacterium interface, and be engineered for size, charge, and surface functionalization to enhance retention within biofilm matrices [236,237]. Positively charged nanoparticles exploit electrostatic interactions with the negatively charged biofilm matrix, improving adhesion and depth of penetration [238,239]. Beyond serving as delivery vehicles, certain metal-based nanoparticles provide intrinsic antibiofilm activity through sustained metal ion release that destabilizes EPS components and through ROS generation that induces oxidative damage to bacterial membranes and intracellular targets [240,241]. Some nanoparticle platforms additionally modulate host immune responses, enhancing macrophage activity and bacterial clearance, thereby extending their therapeutic impact beyond direct bacterial killing [242].

Despite the strong mechanistic rationale, the principal barrier to clinical translation is not antimicrobial efficacy but predictable safety and reproducibility. Metal nanoparticles can induce cytotoxicity, genotoxicity, and off-target oxidative stress, while interactions with biological fluids may promote aggregation, protein corona formation, or exaggerated inflammatory responses that compromise both efficacy and biocompatibility [243,244]. These limitations argue against viewing nanotechnology as a universal systemic therapy and instead support its positioning as a bridge or enabling strategy, most compelling when applied to well-defined clinical bottlenecks, such as localized device-associated infections, topical, or wound delivery, and inhaled formulations for pulmonary disease. In these contexts, careful material design and dosing optimization may allow nanotechnology to enhance antimicrobial exposure while maintaining an acceptable safety profile

## Photodynamic therapy

Photodynamic therapy (PDT) is attractive as a non-antibiotic approach because it bypasses classical antimicrobial targets and instead induces ROS – mediated damage, thereby limiting cross-resistance and directly destabilizing biofilm structures [245]. Across different photosensitizers, therapeutic efficacy is determined less by compound identity alone than by photochemical yield and effective matching between light wavelength and photosensitizer absorption. Riboflavin- and chlorophyllin-based systems consistently disrupt *A. baumannii* biofilms through ROS-dependent mechanisms [246], while methylene blue demonstrates superior antibiofilm activity compared with some synthetic alternatives under comparable conditions [247]. Similarly, radachlorin combined with LED irradiation induces marked morphological disruption of mature biofilms, reinforcing PDT’s capacity to act on established biofilm structures rather than only planktonic cells [248]. The enhanced efficacy observed with combination formulations, such as erythrosine B with acetic acid and chitosan, further indicates that local microenvironment modulation can substantially potentiate PDT outcomes [249].

From a translational perspective, PDT is best suited for localized and accessible infections, including wounds, surfaces, and device-adjacent sites, where controlled light delivery and sufficient oxygen availability can be ensured. Natural photosensitizers such as curcumin and quercetin, along with nano-enabled delivery systems, improve photosensitizer stability and cellular uptake, and extend PDT effects to transcriptional suppression of biofilm-associated genes [250–252]. Mechanistically, PDT not only reduces biofilm biomass but can also re-sensitize embedded bacteria to conventional antibiotics, positioning it as a rational adjunctive strategy rather than a stand-alone systemic therapy for *A. baumannii* infections.

## Phage therapy

Bacteriophage therapy represents one of the most mechanistically aligned non-antibiotic strategies for managing MDR and XDR *A. baumannii*, as phages directly lyse bacterial cells and actively disrupt biofilm communities [253]. Robust efficacy has been demonstrated across pulmonary, wound, and systemic infection models, underscoring their broad applicability in biofilm-dominated disease contexts [254,255]. In respiratory infections, lytic phages significantly reduce bacterial burden and inflammation, while maintaining tissue integrity, and the development of stable inhalable phage formulations further supports translational relevance in pneumonia and ventilator-associated infections [256,257]. Antibiofilm activity in epithelial and lung models strengthens the case for chronic and device-associated respiratory infections [258]. Similarly, in wound and soft tissue infections, dose-dependent antibiofilm activity and improved healing outcomes, particularly when phages are delivered via hydrogels or other localized platforms, highlight their suitability for environments where biofilm persistence is a dominant barrier to treatment [257,259–261].

From a translational perspective, the effectiveness of phage therapy is maximized when three conditions are met: appropriate host-phage matching, anticipation, and management of resistance evolution, and strategic exploitation of phage-antibiotic synergy [262]. Combination therapies consistently outperform phage or antibiotic monotherapies in biofilm clearance, reflecting complementary mechanisms of action and reduced likelihood of treatment failure [263–265]. Importantly, systemic infection models demonstrate not only survival benefits but also the emergence of phage-resistant mutants with reduced fitness, suggesting exploitable evolutionary trade-offs rather than insurmountable resistance barriers [254]. Accordingly, phages are best framed as precision antibacterials whose principal limitations are logistical, such as standardization, regulatory pathways, and scalable manufacturing, rather than biological feasibility. When integrated into rational, mechanism-informed treatment frameworks, phage therapy offers a highly targeted and adaptable approach for controlling multidrug-resistant *A. baumannii* infections.

## Cell-free supernatants

Cell-free supernatants (CFS), including lyophilized CFS (LCFS), represent a promising but still exploratory non-antibiotic approach against *A. baumannii*, distinguished by the combined presence of antibiofilm and immunomodulatory activities. *in vitro* studies demonstrate that CFS can significantly reduce biofilm formation and disrupt pre-established biofilms, accompanied by downregulation of key biofilm-associated genes, such as *bap*, indicating interference with adhesion and biofilm maturation rather than simple growth inhibition [266–268]. This gene-level modulation suggests that CFS targets regulatory pathways central to biofilm development and persistence. In addition, the reported antioxidant activity and compatibility with host immune cells, evidenced by lack of cytotoxicity in RAW 264.7 macrophages and attenuation of nitric oxide production, are particularly relevant in the context of *A. baumannii* infections, which are often associated with excessive inflammatory damage in critical care settings [269–272].

Despite these encouraging findings, the translational readiness of CFS remains limited when compared with more mature strategies, such as antibiotic combinations, phage therapy, or photodynamic therapy. Key barriers include the absence of standardized preparation protocols, incomplete identification of active components, and a lack of pharmacokinetic and pharmacodynamic data defining effective concentrations at infection sites. Moreover, *in vivo* validation in established biofilm infection models is sparse, and potential synergy with antibiotics or other non-antibiotic modalities has not been systematically explored. Accordingly, CFS is best positioned as a hypothesis-generating adjunctive strategy, with future research priorities spanning mechanistic characterization, formulation optimization, *in vivo* efficacy testing, and integration into combination therapies aimed at weakening biofilm integrity and enhancing overall treatment outcomes [273,274].

## Phytochemicals

Phytochemicals are best positioned as multi-target adjuncts rather than stand-alone antimicrobials, as they can simultaneously disrupt bacterial membranes, interfere with biofilm regulatory programs, and attenuate virulence without relying on a single molecular target. In Gram-negative pathogens, such as *A. baumannii*, membrane-destabilizing activity and increased permeability are particularly valuable because the outer membrane constitutes a major barrier to antibiotic entry, thereby enabling synergistic interactions with conventional antibiotics [275,276]. Their most compelling relevance to *A. baumannii* lies in antibiofilm activity: multiple studies demonstrate downregulation of core biofilm-associated genes and disruption of extracellular polymeric substance (EPS) production, directly targeting persistence and horizontal gene transfers within clinical environments [190,277–281].

Despite broad mechanistic promise, the translational impact of phytochemicals is constrained by substantial pharmacological and formulation challenges [282–284]. Variability in compound composition, limited bioavailability, and uncertain dose feasibility at infection sites complicate reproducibility and clinical translation [263,285]. Consequently, the most realistic pathway to clinical relevance is not phytochemical monotherapy but integration into delivery-enabled and synergy-driven frameworks, such as nano- or liposomal formulations and combination regimens that lower antibiotic MICs or disrupt biofilms prior to antibiotic exposure [286,287]. Within this context, phytochemicals function as resistance-attenuating and biofilm-weakening agents that complement, rather than replace, conventional antimicrobial strategies.

## Antimicrobial peptides

AMPs are particularly attractive for *A. baumannii* infections because they retain activity against mature biofilms and disrupt multiple bacterial survival pathways simultaneously, rather than acting through a single molecular target [288,289]. Studies on peptides, such as Octopromycin and Cec4, demonstrate that AMP activity extends beyond membrane permeabilization to include the reprogramming of metabolic and regulatory networks essential for biofilm maintenance [290–293]. This system-level interference likely underpins their efficacy against biofilm-embedded populations that are typically refractory to conventional antibiotics. Notably, LL-37 exhibits antibiofilm activity at concentrations below its minimum inhibitory concentration, indicating that antivirulence or biofilm-disruptive effects may be achievable without maximal bactericidal pressure, a feature that could reduce selective pressure for resistance [294]. Complementary metabolomic analyses, as observed with KHS-Cnd, and the resistance-sparing behavior of Bac7(1–35) further support AMPs as flexible anti-persistence agents capable of destabilizing biofilm-associated metabolic states [295–297].

AMPs are most promising as adjunctive therapies for biofilm-dominated infections, including device-associated infections, wounds, and pulmonary disease, where conventional antibiotics fail to achieve adequate exposure [298]. Their principal barriers to clinical deployment are not mechanistic feasibility but physicochemical and pharmacological challenges, particularly peptide instability, host toxicity at higher doses, and inefficient delivery to infection sites. Addressing these limitations will likely require formulation strategies such as peptide engineering, controlled-release systems, and combination regimens that exploit AMP-mediated biofilm disruption to enhance antibiotic efficacy rather than relying on AMP monotherapy.

## Immunotherapy

Immunotherapy reframes the management of *A. baumannii* infections by shifting the emphasis from direct bacterial killing to enhancement of host-mediated clearance and mitigation of infection-associated damage, thereby potentially reducing selective pressure for antibiotic resistance [299]. Vaccine-based strategies, including multi-epitope formulations and emerging mRNA platforms, are particularly attractive for their capacity to induce durable and strain-transcending immune responses, and may also promote trained innate immunity that accelerates early pathogen control [300,301]. In parallel, monoclonal antibodies provide immediate, antigen-specific passive immunity and have demonstrated the ability to synergize with last-line antibiotics, offering a dual benefit of enhanced bacterial clearance and antibiotic-sparing effects while modulating excessive inflammatory responses [302,303].

Despite this promise, the translational trajectory of immunotherapy remains constrained by antigenic heterogeneity among clinical isolates and the substantial gap between preclinical efficacy and clinical deployment [304]. Manufacturing scalability, cost, and the need for robust clinical validation further complicate implementation. Accordingly, immunotherapy is best positioned as a medium-term strategic pillar, with particular relevance for high-risk settings, such as intensive care units and outbreak-prone healthcare environments, where prevention and early intervention can have an outsized impact. Continued advances in immunoinformatic, antigen discovery, and biologic engineering will be critical in determining whether immunotherapeutic approaches progress from promising adjuncts to practice-changing interventions against multidrug-resistant *A. baumannii* [305,306].

## Clinical evidence and translational landscape of therapeutic strategies

The clinical translation of therapeutic strategies against multidrug-resistant *Acinetobacter baumannii* remains heterogeneous, with most approaches still confined to preclinical evaluation and only a limited number supported by human clinical data. Among available interventions, rational antibiotic combinations demonstrate the most immediate clinical relevance, although evidence remains inconsistent. *in vitro* and observational studies report strong synergistic activity for combinations, such as colistin-doripenem and cefiderocol-ceftazidime/avibactam against MDR and XDR strains [307,308–316]. However, clinical outcomes are variable, for instance, in critically ill ICU patients with carbapenem-resistant *A. baumannii*, the addition of meropenem to colistin plus ampicillin – sulbactam reduced 28-day mortality without significantly improving overall hospital outcomes, highlighting the complexity of translating *in vitro* synergy into clinical benefit. Similarly, observational data suggest that monotherapy, particularly colistin-based regimens, may reduce mortality and adverse events in certain settings, underscoring the need for patient-specific therapeutic optimization.

In contrast, most alternative and adjunctive strategies remain predominantly supported by preclinical evidence. Drug repurposing approaches, including antifungal agents such as itraconazole and fluconazole, metal-based compounds like KP46, and phenothiazine derivatives, have demonstrated antibiofilm activity and the ability to restore antibiotic susceptibility *in vitro*, but lack direct clinical validation [317–323]. Similarly, nanotechnology-based delivery systems, including PLGA, silver, and chitosan nanoparticles, enhance antimicrobial penetration and biofilm disruption in experimental models, yet remain at the preclinical stage due to concerns regarding safety, reproducibility, and regulatory complexity [244,314,324–327]. Photodynamic therapy (PDT) also demonstrates strong *in vitro* efficacy against both planktonic and biofilm-associated cells, particularly when combined with nanomaterials, but has not yet progressed to clinical trials [247,328–331].

Among emerging strategies, bacteriophage therapy represents one of the few approaches transitioning toward clinical application. Early-phase human trials, such as the evaluation of the TP-102 phage cocktail in diabetic foot infections, have demonstrated favorable safety profiles, the absence of treatment-related adverse events, and substantial bacterial reduction, with preliminary evidence of improved wound healing outcomes [332–334]. These findings highlight the potential of phage therapy as a targeted and biofilm-active intervention, although larger randomized trials are required to establish efficacy and standardization. In contrast, other approaches, such as cell-free supernatants, phytochemicals, antimicrobial peptides (AMPs), and immunotherapy remain largely confined to experimental and animal models, despite demonstrating promising antibiofilm, antimicrobial, and immunomodulatory properties [314,334–337].

This disparity highlights the need for well-designed clinical trials, improved pharmacokinetic and pharmacodynamic characterization, and the development of integrated therapeutic frameworks that combine antibiotic and non-antibiotic strategies. Bridging this translational gap will be essential for advancing these interventions from experimental models to standardized clinical practice in the treatment of multidrug-resistant *A. baumannii*. Table 5 summarizes the clinical development status, key findings, and translational evidence of current therapeutic strategies against *A. baumannii*.

**Table 5.** Clinical development status and translational evidence of therapeutic strategies against *Acinetobacter baumannii.*

Therapeutic Approach	Status	Findings	Translational Insight	References
Rational Antibiotic Combinations	Limited clinical trials; supported by observational studies and emerging RCTs	Synergistic activity observed for colistin-doripenem and cefiderocol-ceftazidime/avibactam against MDR/XDR strains. In ICU patients with CRAb, adding meropenem to colistin + ampicillin-sulbactam reduced 28-day mortality, though overall outcomes were unchanged. Monotherapy (e.g. colistin-based) may reduce adverse events in some cases	Most clinically relevant strategy; effectiveness depends on PK/PD optimization, infection severity, and patient-specific factors	[307,308–316]
Drug Repurposing	Preclinical; no clinical trial data	Agents such as itraconazole, fluconazole, KP46, and phenothiazines exhibit antibiofilm activity and can restore antibiotic susceptibility	Promising adjunctive strategy targeting non-classical pathways; limited by lack of clinical validation and dose feasibility	[317–323]
Nanotechnology/Nano-Delivery Systems	Preclinical stage; no clinical trials	Nanoparticles (PLGA, silver, chitosan) enhance drug delivery, disrupt biofilms, and improve bactericidal activity; potential for targeted delivery (e.g. intracranial infections)	Enables improved antimicrobial penetration and retention; limited by safety, scalability, and regulatory challenges	[244,324–327]
Photodynamic Therapy (PDT)	Preclinical; no clinical trials	Photosensitizers (riboflavin, chlorophyllin, methylene blue) effective against planktonic and biofilm cells; enhanced by nanomaterial integration	Effective localized therapy; limited by light penetration and lack of clinical standardization	[247,328–331]
Phage Therapy	Early-phase clinical trials; ongoing preclinical studies	TP-102 phage cocktail showed safety, no adverse effects, and significant bacterial reduction (80% vs 50% placebo) with improved wound healing trends	Most advanced non-antibiotic strategy; requires optimization of host-phage matching, delivery, and regulatory pathways	[332–334]
Cell-Free Supernatants/Probiotic-Derived Products	No clinical data reported	Limited or no available data	Experimental concept; requires mechanistic characterization and *in vivo* validation	[nr]
Phytochemicals	Preclinical evidence only	Plant-derived compounds (e.g. *Myrtus communis*) show antibacterial activity and antibiotic synergy	Adjunctive anti-biofilm strategy; limited by bioavailability and reproducibility	[335]
Antimicrobial Peptides (AMPs)	Preclinical stage; no clinical trials	Strong antibiofilm and bactericidal activity against MDR strains	Promising alternative or adjunct; limited by stability, toxicity, and delivery challenges	[332,334,335]
Immunotherapy	Preclinical stage; no clinical trials	Nanoparticle-based delivery of outer membrane proteins induces protective immunity in animal models	Preventive and adjunctive potential; limited by antigen variability and lack of clinical validation	[312]

### Knowledge gaps and Future research directions

Significant knowledge gaps remain in addressing infections caused by *A. baumannii*, particularly in translating emerging technologies into clinical practice. Advances in computational biology and systems-level approaches have enabled high-throughput screening for novel drug targets and lead compounds, yet their integration with experimental and clinical validation remains limited [338] (Skariyachan et al., 2019). Future directions should prioritize combination therapies, especially the use of anti-virulence agents alongside existing antibiotics, to enhance therapeutic efficacy while mitigating resistance development [339]. In parallel, strengthened global surveillance and multinational collaboration are essential to monitor resistance trends, track high-risk clones, and inform targeted interventions [336]. Additionally, artificial intelligence and deep learning are increasingly being explored to optimize drug discovery pipelines and improve infection control strategies, although challenges in interpretability and clinical integration persist [337]. Collectively, these approaches underscore the need for multidisciplinary, translational frameworks to effectively combat extensively drug-resistant *A. baumannii*.

## Conclusion

*A. baumannii* has transitioned from a pathogen of limited concern to a critical global threat because of its exceptional genetic adaptability, capacity to accumulate diverse resistance determinants, and reliance on QS – regulated biofilm formation to promote persistence, virulence, and antimicrobial tolerance. The convergence of multidrug resistance, biofilm-mediated protection, and efficient horizontal gene transfer has progressively undermined the effectiveness of conventional therapies, particularly against carbapenem-resistant strains designated as critical priority pathogens by the WHO. Although last-line antibiotics remain clinically indispensable, their declining efficacy underscores the limitations of treatment paradigms centered exclusively on traditional antimicrobial agents. Addressing the *A. baumannii* challenge therefore requires a paradigm shift toward mechanism-informed and integrative therapeutic strategies. Advances in understanding resistance networks, quorum sensing, and biofilm biology provide a rational foundation for combining optimized antibiotic regimens with delivery-enabled approaches and non-antibiotic modalities that disrupt biofilms, attenuate virulence, or enhance host-mediated clearance. Importantly, no single intervention is likely to be sufficient. Durable control will depend on coordinated, multifaceted frameworks that integrate novel drug development, adjunctive and alternative therapies, and robust infection prevention and stewardship practices. Without such alignment between molecular insight and clinical strategy, the continued expansion of extensively drug-resistant *A. baumannii* threatens to further erode the effectiveness of modern antimicrobial therapy.

## Abbreviations

AACs: Aminoglycoside acetyltransferases
AHL: acyl homoserine lactone
AMEs: aminoglycoside-modifying enzymes
AMP: antimicrobial peptides
AMR: antimicrobial resistance
ANTs: Aminoglycoside nucleotidyltransferases
APHs: Aminoglycoside phosphotransferases
ATP-PRT: ATP-phosphoribosyltransferase
Bap: biofilm-associated protein
CCCP: carbonyl cyanide m-chlorophenylhydrazone
CFS: cell-free supernatant
CLSM: confocal laser scanning microscopy
CRAb: Carbapenem-resistant *Acinetobacter baumannii*
Csu: chaperone-usher pili
EP: Extracellular polysaccharide
EPS: extracellular polymeric substances
FDA: Food and Drug Administration
GaN: gallium nitrate
LCFS: lyophilized CFS
LED: Light-Emitting diode
LPS: lipopolysaccharide
MATE: multidrug and toxic compound extrusion
MBC: minimum bactericidal concentration
MBLs: metallo-beta lactamases
MDR: multidrug-resistant
MFS: major facilitator superfamily
MICs: minimum inhibitory concentrations
OmpA: outer membrane protein A
PBPs: penicillin-binding proteins
PDR: pan-drug-resistant
PDT: Photodynamic therapy
pEthN: phosphoethanolamine
PMQR: plasmid-mediated quinolone resistance
PNAG: poly-β-1,6-N-acetylglucosamine
QRDRs: quinolone resistance – determining regions
QS: quorum sensing
RND: resistance – nodulation – division
ROS: Reactive oxygen species
SEM: scanning electron microscopy
SMR: small multidrug resistance family
XDR: extensively drug-resistant
